# Anomaly Field Extraction Based on Layered-Earth Model and Equivalent Eddy Current Inversion: A Case Study of Borehole zk506 in Baishiquan, Xinjiang

**DOI:** 10.3390/s25113502

**Published:** 2025-06-01

**Authors:** Yi Yang, Jie Zhang, Qingquan Zhi, Yang Ou, Xingchun Wang, Lei Wang, Junjie Wu, Xiaohong Deng

**Affiliations:** 1 State Key Laboratory of Deep Earth Exploration and Imaging, The Institute of Geophysical and Geochemical Exploration, CAGS, Dongli, Tianjin 300300, China; yyi@mail.cgs.gov.cn (Y.Y.); oyang@mail.cgs.gov.cn (Y.O.); wxingchun@mail.cgs.gov.cn (X.W.); wjunjie@mail.cgs.gov.cn (J.W.); dengxiaohong@mail.cgs.gov.cn (X.D.); 2 Laboratory of Geophysical EM Probing Technologies, MLR, Dongli, Tianjin 300300, China; 3 The Institute of Geophysical and Geochemical Exploration, CAGS, Dongli, Tianjin 300300, China; 4 No.6 Geological Survey Team, Bureau of Xinjiang Geological Exploration, Hami 839000, China; wanglei@mails.cgs.gov.cn

**Keywords:** BHTEM, equivalent eddy current, anomaly field extraction, pure anomaly

## Abstract

Based on the concept of an equivalent eddy current, anomaly field inversion provides an efficient and rapid inversion method for borehole transient electromagnetic (BHTEM) measurements. It enables the utilization of the equivalent eddy current to rapidly process and interpret BHTEM data. This method allows for the accurate determination of the central position and spatial distribution of anomalies. However, the equivalent eddy current method is solely applicable to the inversion of the anomaly field. Given that the measured data frequently contain strong background field information, it is challenging to directly apply the equivalent eddy current approach to the inversion and interpretation of the measured data. To address the aforementioned issues, in this study, we innovatively put forward a method. Specifically, we utilize the response of the layered earth to simulate the background field and subtract the background field from the measured data through the “difference method” to extract the anomaly field. Subsequently, by integrating the equivalent eddy current method, the inversion of the anomaly field was accomplished. Eventually, a rapid quantitative inversion and interpretation of BHTEM data were achieved. We applied this approach to extract the pure anomaly from the measured data of the zk506 borehole in the Baishiquan mining area, Xinjiang, and then conducted equivalent eddy current inversion. The spatial position and distribution characteristics of the concealed ore bodies near the zk506 borehole were successfully pinpointed. Validation by the zk507 and zk508 boreholes confirmed that the main anomaly of the nickel ore body is positioned in the southeast of the boreholes, dipping northwestward. This outcome validates the accuracy of the BHTEM inversion interpretation and rectifies the geological understanding obtained from the zk506 single borehole. It demonstrates the effectiveness and significance of the pure anomaly extraction based on the layered-earth model and equivalent eddy current inversion in the exploration of high-conductivity sulfide ores.

## 1. Introduction

Mineral resources constitute the bedrock for a nation’s economic development. Exploration of these resources is of paramount importance in safeguarding national resource security. At present, with the rapid depletion of shallow mineral deposits, prospecting efforts are gradually shifting to greater depths. When confronted with complex terrains and the need to detect deep-seated ore bodies, traditional surface exploration methods encounter challenges. For instance, issues such as limited accuracy and significant interference arise [1,2,3]. These problems demand higher standards for both the depth and precision of detection [4,5]. Addressing the limitations of traditional surface exploration methods in complex terrains and deep-seated ore body detection, the BHTEM method emerges as a viable solution. Operating on a ground-emission and borehole-reception principle, the BHTEM method [6] offers distinct advantages. Firstly, the earth acts as a natural filter, effectively reducing ground-based interference [7]. Secondly, with the borehole probe in close proximity to anomalies, it captures stronger signals [8]. This approach overcomes the drawbacks of surface-based techniques, providing a precise and in-depth tool for mineral exploration [9]. Over the years, the BHTEM method has proven highly successful in the exploration of sulfide-type copper/nickel deposits [10,11,12,13]. It has become an essential technique for identifying deep-seated and concealed ore bodies, as demonstrated by numerous studies [14,15,16,17]. With the development of artificial intelligence technology, machine learning methods have been increasingly applied to geophysical data processing, significantly enhancing data interpretation capabilities [18,19]. This highlights the importance of new information processing technologies in geophysical exploration [20,21,22]. The primary objective of BHTEM detection is to pinpoint the location of blind ore bodies at the bottom or adjacent to the borehole, thereby providing crucial support for ore body tracing. Currently, there are three main positioning methods. The first method involves determining the anomaly center through vector intersection, leveraging the magnetic field response generated by induced eddy currents [23]. This approach is relatively fast; however, it falls short in discerning the spatial distribution of anomalies. The second method is the equivalent eddy current inversion [24,25,26]. By equating the anomaly response to that of an eddy current, this method can rapidly ascertain the spatial location and distribution of anomalies. Nevertheless, it necessitates obtaining the pure anomaly response of the underground abnormal body. The third method is 3D inversion [27,28,29]. This technique can yield accurate information regarding anomaly location, spatial distribution, and resistivity. However, it involves generating a global mesh, which typically demands substantial computing resources. Additionally, the data from a single borehole is rather sparse in the inversion space, failing to impose effective constraints on the global grid. As a result, the accuracy and uniqueness of the inversion are compromised, making it difficult to achieve satisfactory results in practical applications.

In addressing these challenges, this study presents an innovative technology that integrates anomaly field extraction with equivalent eddy current inversion. Initially, this approach utilizes a layered-earth model to fit the background, effectively isolating the pure response of the underground ore body. Subsequently, the equivalent eddy current method is employed to model this pure response, enabling the precise determination of the location of deep-seated anomalies. This not only represents an advancement in the equivalent eddy current inversion technique but also substantially enhances its practical applicability. Finally, through a comprehensive field case study, this method is applied to the BHTEM-measured data. It successfully predicted the spatial location and distribution characteristics of the ore body, and the results were validated through drilling.

## 2. Theoretical Methods and Case Studies Verification

### 2.1. Equivalent Eddy Current Theory

**Figure 1:**
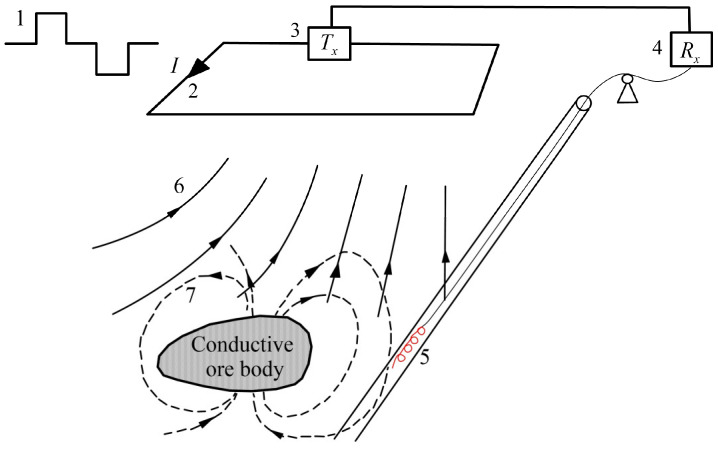
**Schematic diagram of BHTEM.** 1. Transmission waveform; 2. transmission loop, current direction, and magnitude; 3. transmitter (Tx); 4. receiver; 5. borehole receiving probe; 6. transmission loop primary field; 7. secondary field generated by borehole conducting ore body.

The BHTEM method generally adopts a ground-transmitting and borehole-receiving configuration, as depicted in Figure 1. When a step current is applied to the transmitting loop, it generates a primary magnetic field. This primary magnetic field induces a secondary current within the underground conductive rocks or ore bodies. Once the transmitting current is abruptly terminated, the receiving coil captures the time-varying signal of the secondary magnetic field. By meticulously analyzing these signals and applying the principle of electromagnetic induction, which relates the induced electromotive force to the rate of change of the secondary magnetic field, we can infer the resistivity information of geological bodies at different depths underground.

**Figure 2:**
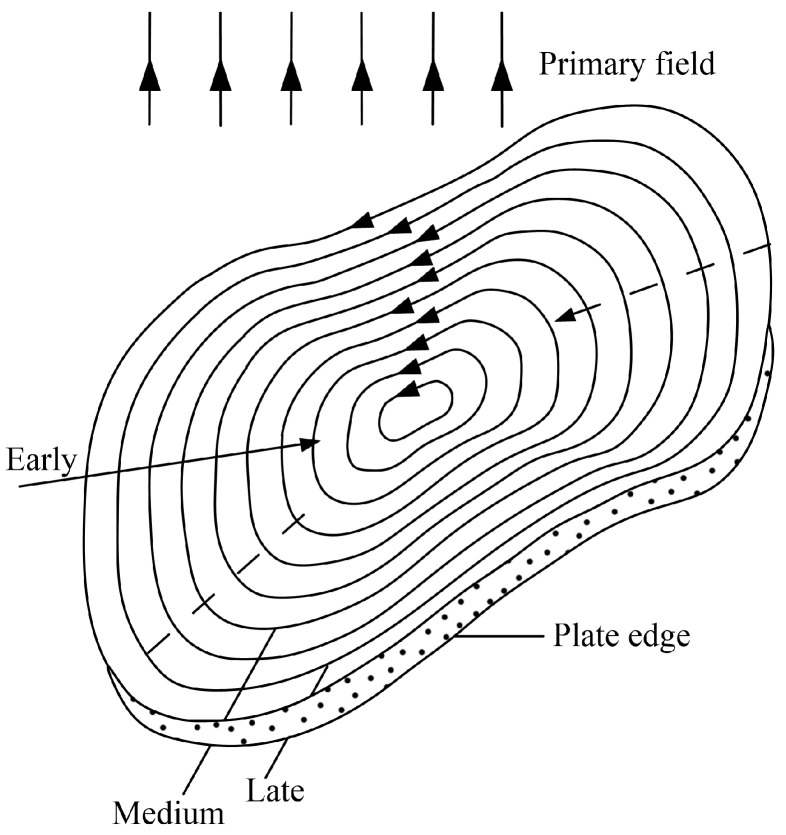
Schematic diagram of eddy current distribution in conductive thin plate (From [18]).

**Figure 3:**
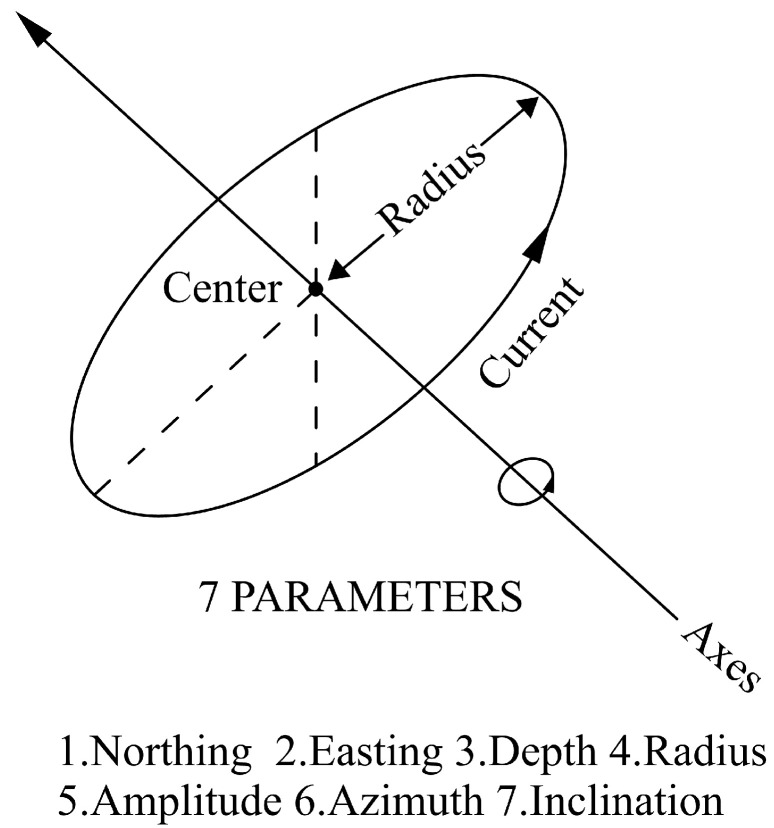
Magnetic field response of equivalent current in free space.

Figure 2 depicts the physical process of induced eddy current generation and dissipation within a conductive thin plate under a primary magnetic field. When a conductive thin plate is placed in a primary magnetic field and the transmitting process suddenly stops, according to Faraday’s law, to maintain the influence of the primary field before shutdown, induced eddy currents will arise within the conductive thin plate. These induced eddy currents form a series of eddy currents inside the plate. Resembling the shape of the plate, these loops vary in intensity and size. Initially, these loops are mainly distributed at the edges of the plate and are relatively large. As time passes, they gradually spread toward the center of the conductor and eventually dissipate due to heat loss. At any moment during this process, the induced current distribution within the conductive thin plate can be approximated by the field generated by an equivalent eddy current [24]. Therefore, the characteristics of a specific conductive thin plate can be described through an equivalent eddy current (Figure 3). This loop, located in free space, can be defined by seven parameters, and its magnetic field in free space can be calculated using Formulas (1) and (2).

(1) $$\;B_{\rho }=\frac{\mu I}{2\pi }\frac{z}{\rho {\left[{\left(a+\rho \right)}^{2}+z^{2}\right]}^{\frac{1}{2}}}\left[-K+\frac{a^{2}+\rho^{2}+z^{2}}{{\left(a-\rho \right)}^{2}+z^{2}}E\right]$$

(2) $$B_{z}=\frac{\mu I}{2\pi }\frac{z}{\rho {\left[{\left(a+\rho \right)}^{2}+z^{2}\right]}^{\frac{1}{2}}}\left[K+\frac{a^{2}-\rho^{2}-z^{2}}{{\left(a-\rho \right)}^{2}+z^{2}}E\right]$$

Among them, $\rho ={\left(x^{2}+y^{2}\right)}^{1/2}$, $B_{\rho }$ is the radial component of the magnetic field, $B_{z}$ is the axial component of the magnetic field, K and E are the first and second types of elliptic integrals, a is the radius of the circular eddy current, μ is the free-space magnetic permeability, and I is the current intensity of the eddy current.

### 2.2. Background Field Calculation

The layered-earth model is employed in the simulation research of the background field. The rectangular-loop transmission source of transient electromagnetics can be approximated by multiple electric dipoles of a certain length. The response of the electric dipoles is derived using Kerry Key’s [30] method. The vector potential generated by a horizontal electric dipole oriented in the x-direction at any point on a layered medium is as follows:

(3) $$\;A_{x}\left(r\right)=\frac{1}{2\pi }\int_{0}^{\infty }{\hat{A}}_{x}(\lambda ,z)J_{0}(\lambda r)\lambda d\lambda$$

(4) $$A_{z}\left(r\right)=\frac{1}{2\pi }\frac{\partial }{\partial x}\int_{0}^{\infty }{\hat{A}}_{z}\left(\lambda ,z\right)J_{0}\left(\lambda r\right)\lambda d\lambda \;\;$$

The corresponding magnetic field components are expressed as follows:

(5) $$\;H_{x}=\frac{\partial A_{Z}}{\partial y}$$

(6) $$H_{y}=\frac{\partial A_{x}}{\partial y}-\frac{\partial A_{z}}{\partial x}$$

(7) $$H_{z}=-\frac{\partial A_{x}}{\partial y}$$

Among them, ${\hat{A}}_{x}$ represents the integration kernel in the x-direction, $A_{x}\left(r\right)$ is the vector potential in the x-direction, ${\hat{A}}_{z}$ is the integration kernel in the z-direction, and $A_{z}\left(r\right)$ denotes the vector potential in the z-direction. $J_{0}$ stands for the Bessel function of the first kind of order zero. $H_{x}$, $H_{y}$, and $H_{z}$ denote the magnetic field strengths in the x, y, and z directions, respectively.

The magnetic field response obtained through the above steps is a complex integral containing Bessel functions, which is solved using Hankel digital filtering calculation [31]. Its general expression can be written as follows:

(8) $$\;G^{\prime }\left(v\right)=\sum_{n=-\infty }^{\infty }F\left(n∆\right)H_{v}^{*}\left(v-n∆\right)$$

Among them, ∆ is the sampling interval, n is the number of filter points, and $H_{v}^{*}$ is the filter coefficient. After completing the calculation of the frequency domain field, the cosine transform is used to convert the field values into the time domain. Taking $H_{z}$ as an example, the numerical discrete approximation expression is written as follows:

(9) $$\;H_{z}\left(t\right)=\frac{2}{\pi t^{2}}\sum_{j=1}^{n}\frac{R_{j}-R_{j-1}}{\omega_{j+1}-\omega_{j}}\left[\cos\left(\omega_{j}t\right)-\cos\left(\omega_{j+1}t\right)\right]$$

Among them, $R=Im\left[H_{z}(\omega )\right]\frac{1}{\omega }$ is the vertical magnetic field, $H_{z}$ is the angular frequency, ωt is the sampling delay, and π is the pi.

Similarly, the electromagnetic field response of a directional dipole can be obtained.

To validate the accuracy of the algorithm, Maxwell(5.4.53.17914), a widely recognized and industry-proven commercial software for transient electromagnetics, was selected for comparison (for detailed information, refer to https://www.electromag.com.au/maxwell.php (accessed on 5 April 2025)). The designed model, as presented in Table 1, consists of five layered-earth models. The transmitting and receiving parameters adopted are as follows: the transmission frame size is 200 m × 200 m, the transmission current is 1A, the transmission time base is 6.25 Hz, the sampling time is Crone 50 ms (there are 36 sampling channels), the rising ramp is 0.00001 ms, the falling ramp is 0.00001 ms (there are 100 measuring points), and the interval is 5 m. Unless otherwise specified, the delay chosen for the numerical simulations below is Crone 50 ms.

**Figure 4:**
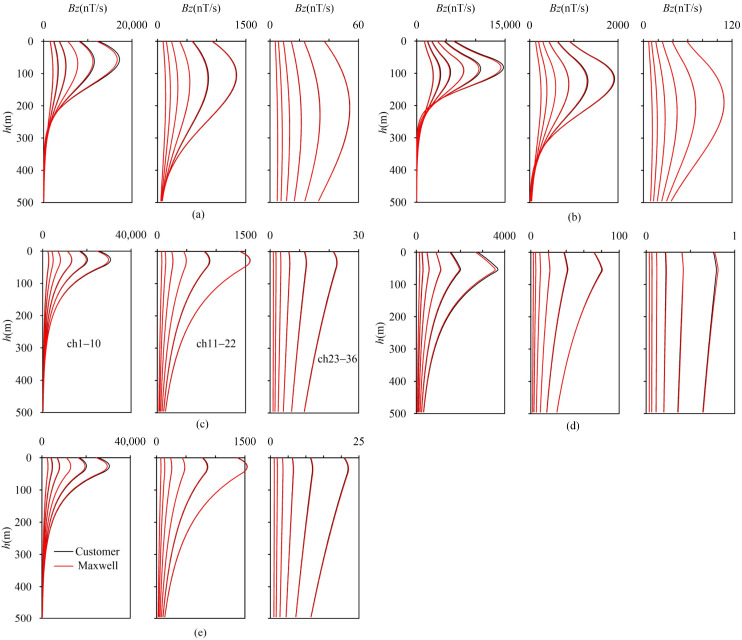
Verification of response curve of layered-earth model. (**a**) A 100 Ω·m uniform half-space; (**b**) D-type; (**c**) G-type; (**d**) H-type; (**e**) Q-type.

As can be observed from Figure 4a–e, the response computed by the self-compiled program and those from the Maxwell lerio module under different layered models are generally in good agreement. With the exception that the amplitude of the first channel shows a slight difference, the other channels are fitted well. Table 2 indicates that the maximum average relative error for each measurement point within the same channel is 3.96%, and the maximum relative error of a single measurement point in a single channel is 4.87%.

### 2.3. Example of Anomaly Field Extraction

In the previous section, the validity of the background field algorithm has been verified. Next, we will integrate anomaly field extraction (background field elimination) with the equivalent eddy current technique to perform an inversion test on the theoretical model, so as to evaluate its applicability.

**Figure 5:**
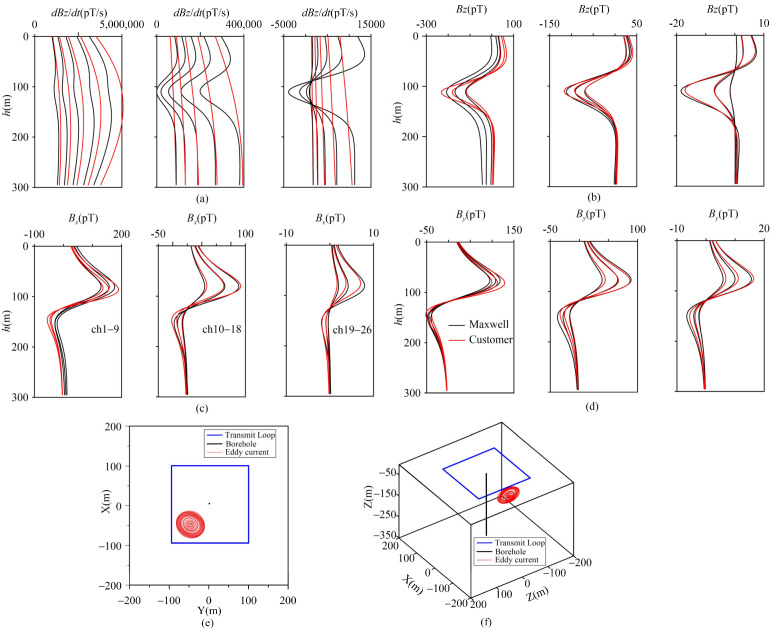
Uniform half-space test model with conductive thin plate. (**a**) Uniform half-space inductance response with conductive thin plate; (**b**) abnormal z-component and equivalent eddy current response; (**c**) abnormal x-component and equivalent eddy current response; (**d**) abnormal y-component and equivalent eddy current response; (**e**) spatial distribution of equivalent eddy current in x-y plane; (**f**) spatial distribution of equivalent eddy current in stereo perspective.

**Figure 6:**
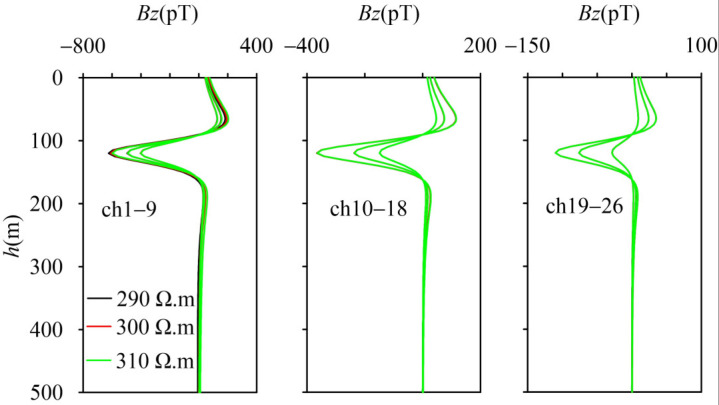
Anomalies extracted by the “difference method” under disturbance conditions.

In this study, we employ the BHTEM response of a conductive thin plate within a uniform half-space as the test data. The resistivity of the uniform half-space is set at 300 Ω·m, the dimensions of the transmitting frame are 200 m × 200 m, and the sampling delay is 50 ms. To be consistent with actual working parameters, the rising ramp is set to 0.25 ms and the falling ramp to 0.5 ms. The conductive thin plate has dimensions of 50 m × 50 m, with its center coordinates at (−50, −50, −100). Its longitudinal conductivity is 500 S, the inclination angle is 25°, and the inclination rotation angle is 30°. Under the same background resistivity, the response obtained through the self-compiled program is larger than that obtained using Maxwell (as shown in Figure 5a). Besides considering that the background half-space and thin plate coupling diminish the overall response, the primary influencing parameter is the falling ramp. During the program algorithm verification phase, the falling ramp was set to 0.00001 ms, indicating an impulse response for the emission waveform. However, when the falling ramp was adjusted to 0.5 ms, significant differences were observed in the early stage between the responses calculated by the self-compiled program and Maxwell (Figure 4, ch1–ch9). This discrepancy arises from the finer segmentation of the falling ramp in our program, where a 0.5 ms falling ramp is divided into 30 calculations, leading to a prolonged computation time. In contrast, Maxwell’s software uses a relatively fast but crude method for calculating falling ramp to increase efficiency. Consequently, the calculation results show discrepancies in the early stage, while the fitting is more precise in the middle stage (as depicted in Figure 6, channels 10–26). It is clearly evident from Figure 5a that the response generated by our self-compiled program effectively depicts the characteristics of the background field of the test model. The abnormal field, obtained via the “difference method”, closely approximates the equivalent eddy current field (Figure 5b–d). The spatial positions of the eddy currents corresponding to different channels after inversion are presented in Figure 5e–f. Significantly, the size of these loops is slightly smaller than that of the conductive thin plate in the Maxwell model. Nevertheless, the central coordinates, inclination, and azimuth, which represent the spatial location, distribution, and shape of the anomaly, are essentially the same. These parameters are regarded as typical, and their average values are tabulated in Table 3.

As can be seen from Table 3, the average values of typical parameters for the eddy current indicate that the anomaly location and spatial distribution align with the thin plate parameters in the designed model. This calculation example demonstrates that the anomaly extraction based on the layered-earth and equivalent eddy current inversion is feasible.

### 2.4. Robustness Evaluation

When simulating the background field for measured data, the selection of parameters is of critical importance. Therefore, it is necessary to evaluate the perturbation caused by minor changes in formation resistivity on the response. We designed three homogeneous half-space models (Table 4) to assess the impact of parameter variations on abnormal field extraction and inversion results by altering the resistivity values of the homogeneous half-space. In the calculation of the model containing a conductive thin plate, the background model is selected as Model 1 in Table 4, and the thin plate model in Section 2.3 is adopted for the thin plate.

Under disturbance conditions, the anomaly extraction results of the “difference method” show that the overall shape of the abnormal values is consistent with the response of the thin plate in free space. The three types of disturbances caused by changing the background resistivity values have limited effects on anomaly extraction, and their impacts only occur in the early stage (Figure 6, channels 1–9), without contributing to the responses in the middle and late stages, and ultimately have almost no impact on inversion. This also demonstrates that the anomaly extraction based on the “difference method” has good robustness. Of course, since transient electromagnetic (TEM) is sensitive to low-resistivity bodies, this method needs to be used with caution in the presence of low-resistivity backgrounds.

## 3. Baishiquan BHTEM Survey

### 3.1. Geological Background

The strata exposed in the Baishiquan copper/nickel mining area consist of an ancient metamorphic rock series. These ancient basement rocks have undergone numerous tectonic movements, which provide essential conditions for the formation of ore deposits in this area. The Baishiquan copper/nickel mine is located 3 km south of the Shaquanzi fault, where neutral, basic, and ultrabasic rocks occupy the fault zones, serving as primary conduits for magmatic activity in the area [32,33]. The ore-bearing rock mass consists of a basic–ultramafic complex, characterized by an elliptical lithological unit at its core as the predominant component (Figure 5a). The eastern contact zone displays an arcuate distribution, dipping from northwest to southwest and forming an inward-steepened basinal structure. Copper/nickel mineralization is predominantly hosted within ultramafic lithologies of the complex [34], which show peripheral concentration with minor intra-complex occurrences. Principal lithological units comprise pyroxenite, olivine pyroxenite, plagioclase peridotite, and lherzolite, with lherzolite demonstrating optimal ore potential. To date, 28 copper/nickel ore bodies have been identified in the Baishiquan deposit, the most significant being designated as Ni1, Ni12, and Ni13 [35]. These ore bodies display lenticular, stratoid, and vein-type morphologies, characterized by distinct branching/recombination structures (Figure 5b). Most ore bodies exhibit surface exposure or subcropping features attributed to shallow hypogene positions. Dimensionally constrained, the ore bodies range from 10 to 200 m in strike length (locally > 300 m), with thicknesses averaging 1–15 m (maximum > 15 m).

**Figure 7:**
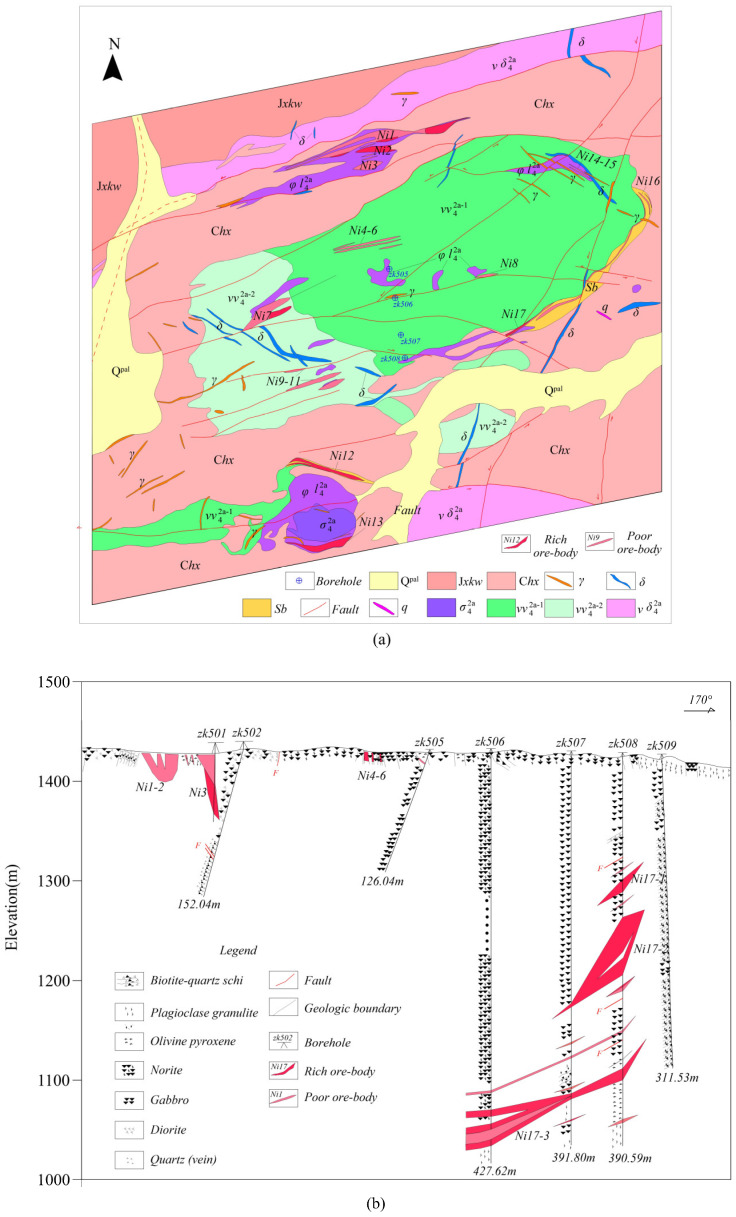
Schematic diagram of Baishiquan area. (**a**) Geological background of Baishiquan mining area (modified according to [34]); (**b**) zk506 exploration line geological drilling profile.

**Figure 8:**
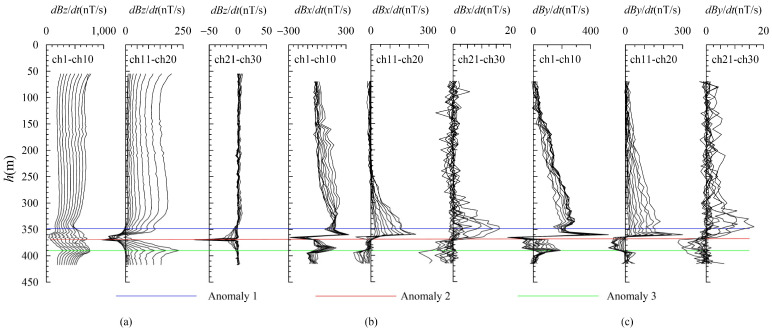
Zk506 BHTEM response. (**a**) Z-component response; (**b**) x-component response; (**c**) y-component response.

To evaluate the resource potential systematically, mineral exploration entities have deployed exploration boreholes in the central sector of the mining district, including borehole ZK506 (Figure 7a). Drilling intersected three mineralized intervals of copper/nickel sulfide mineralization. Lithostratigraphic logging delineates a vertical sequence comprising gabbroic norite, mineralized peridotite, and plagiogranite, with the gabbroic norite recurrently intercalated within the sequence. Significant stratiform mineralization occurs specifically at the petrologic contact between gabbroic norite and plagiogranite. Preliminary structural analysis indicates a southeastward dip of the ore zone (Figure 8a, uncorrected geological profile). In order to delineate the spatial configuration and axial geometry of the geophysical anomaly associated with borehole ZK506, BHTEM surveys were conducted within the drill hole.

### 3.2. BHTEM Data Acquisition

Data acquisition was conducted utilizing a 2.4 kW Digital Pulsed Electromagnetic (PEM) system configured with the following parameters: emission current maintained at 15 A, a time base duration of 20 ms, and a turn-off time of 500 μs. The transmitter system utilizes a central loop configuration of 100 m × 100 m, with the borehole collocated at the geometric center of the loop.

It is evident that there are three distinct abnormal manifestations in the z-component (as shown in Figure 8). These are located at the positions marked by the blue horizontal line at 340 m, the red horizontal line at 365 m, and the green line at 390 m. By analyzing the morphology and amplitude of these anomalies in the early, middle, and late periods, it becomes clear that each of these three anomalies has its own unique characteristics.

The anomaly marked by the blue horizontal line is a low-amplitude negative anomaly, likely indicating a small-scale or low-grade ore body near the borehole. The red-marked anomaly initially shows a small positive amplitude before transitioning to extremely strong negative values in mid-to-late stages, interpreted as a borehole anomaly intersecting the ore head. Its intense mid-to-late-stage response, especially the extremely strong negative anomaly in the late stage, suggests a large-scale or high-grade ore body near the borehole. The green horizontal line marks a positive anomaly with strong early-to-medium-stage signals (weaker than the red anomaly) but no late-stage response, identified as an in-hole anomaly with the borehole position essentially centered within it, indicating a medium-scale or low-grade ore body.

At the depth of 370 m, the negative anomaly response of the z-component indicates the presence of a large-scale or high-grade blind ore body near the borehole. Combined with the characteristics of the x- and y-component curves, this anomaly is determined to be located in the third quadrant. Additionally, the positive response values at one end of the zero point of the horizontal component along the reverse “S”-shaped curve are all greater than the negative response values, suggesting a tendency for this anomaly to tilt toward the west and north directions.

### 3.3. Pure Anomaly Extraction and Equivalent Eddy Current Inversion

Based on the geological logging of borehole zk506, the lithology and thickness of the formation can be ascertained. Regarding the formation resistivity value, it draws reference from the copper/nickel mining area in the east of Huangshan, which has similar metallogenic conditions (Table 5).

**Figure 9:**
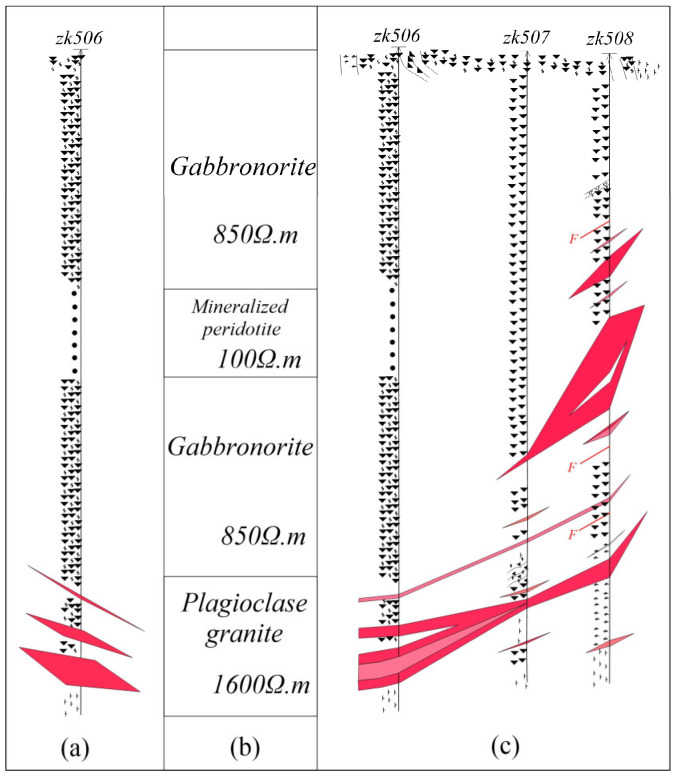
Borehole zk506’s geology and resistivity stratification. The meaning of the symbols in the figure is consistent with that in Figure 7b. (**a**) The ore body strike is predicted according to single-hole geological logging; (**b**) the layered-earth resistivity model is given according to the formation lithology and electrical parameters; (**c**) ore body strike and extension controlled by multiple boreholes.

**Figure 10:**
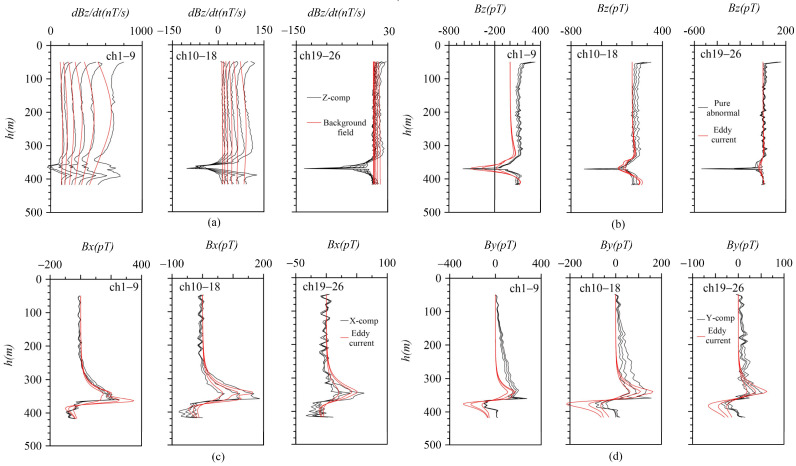
Zk506 BHTEM data anomaly extraction and equivalent eddy current inversion fitting curve. (**a**) Z-component and background field fitting curve; (**b**) z-component pure anomaly and eddy current fitting curve; (**c**) x-component and eddy current fitting curve; (**d**) y-component and eddy current fitting curve.

Based on the statistical values of the background resistivity of rocks and ores, the initial formation background thickness and resistivity values were provided (Figure 9b). Through man–machine interactive trial calculations, the formation resistivity was appropriately adjusted until the fitting of the background field met the requirements (Figure 10a). Then, the background field was subtracted from the measured data to obtain the pure anomaly field (Figure 10b). Based on the morphological characteristics of BHTEM-measured curves and borehole logging, three main anomalies can be identified in this borehole. Among them, the primary anomaly occurs at 370 m depth, located in the southeast direction of the borehole. The anomaly at 340 m exhibits the smallest amplitude and is positioned northeast of the borehole. The 390 m anomaly represents an in-borehole anomaly with a depth range between 340 m and 370 m. Generally, the eddy current radius should be smaller than the transmitting frame radius, and the current intensity should be set to relatively low values. The inclination and rotation angles are determined according to curve morphology [26]. The initial parameters of the given eddy current are detailed in Table 6.

## 4. Discussion

**Figure 11:**
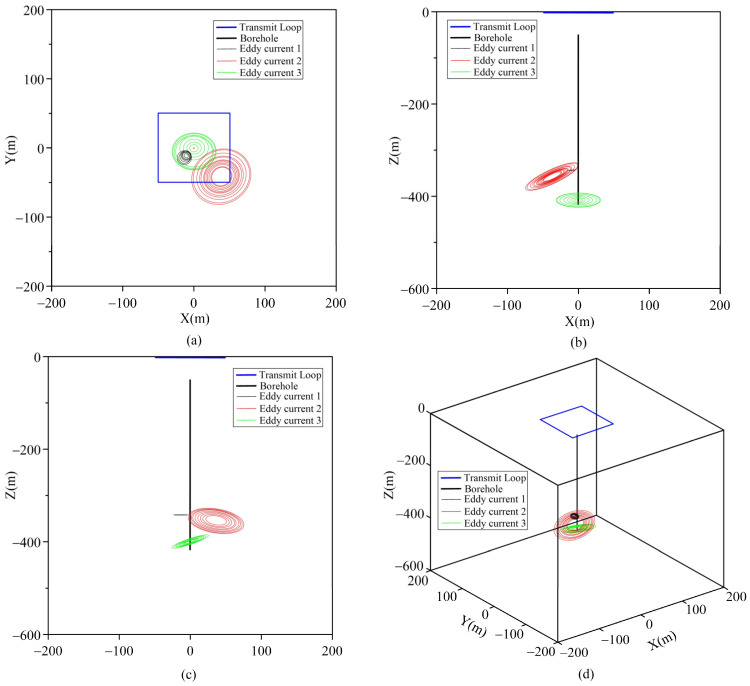
Spatial distribution of the equivalent eddy current in borehole zk506. (**a**) The x-y plane distribution of eddy current; (**b**) x-z plane distribution of eddy current; (**c**) y-z plane distribution of eddy current; (**d**) three-dimensional distribution of eddy current.

From the fitting curves of each component (Figure 10a), it is evident that the early-stage curves following anomaly extraction exhibit varying degrees of distortion. In contrast, the middle- and late-stage curves are minimally influenced by background factors and can effectively reflect the true anomaly. Consequently, the central coordinates and two angle parameters associated with the middle- and late-stage anomalies carry greater practical significance. These parameters have been systematically tabulated in Table 7 for clarity and reference. Figure 11a–d show the spatial distribution characteristics of the eddy current at different angles.

It can be observed from Figure 9b that, after the extraction of the anomaly field, an anomaly with a narrow opening and large amplitude appears at 370 m on the z-component curve. This suggests that the anomaly exhibits significant extension along the inclined direction. The circular eddy current model fails to adequately simulate this anomaly, resulting in the inability to fit the z-component at its maximum value, although the other components are well fitted. When combined with the shape analysis of the x- and y-component curves, it was determined that the main anomaly (marked by the red horizontal line in Figure 8) was positioned in the southeast direction of the borehole. However, the interpretation of these anomalies based on the survey results of BHTEM does not conform to the geological logging of the zk506 borehole at that time. According to the drilling outcomes of the zk506 borehole, geologists at that time believed that the anomalies had a gentle inclination toward the southeast and were nearly horizontal (Figure 9a). Through the logging of the zk507 and zk508 boreholes constructed in the subsequent stage, geologists recognized that the main ore body was located in the southeast direction of the zk506 borehole and inclined toward the northwest (Figure 7b). This finding was consistent with the ore body tendency and orientation inferred by the BHTEM, thus demonstrating the significance and necessity of developing the BHTEM method in the exploration of sulfide copper/nickel ore deposits.

The late response of the z-component of the measured data is strong and sharp. Considering that it is caused by the uneven conductivity and long extension of the local anomaly, the thin plate can basically reflect the spatial distribution of the anomaly. Figure 8a presents the original logging details of borehole zk506. At that time, there were three ore layers. The first layer was lean ore, while the second and third layers were high-grade ore. Prior to conducting BHTEM measurements, geological personnel considered Layer 3 to have considerable thickness and predicted it to be the primary ore layer, determining that the ore body exhibited a gently inclined southeastward attitude. After the BHTEM survey, based on the characteristics of the z-component response curve, it is inferred that the extremely strong Anomaly-2 is the main anomaly (Figure 8) which suggests that there is a large-scale or high-grade blind ore in the vicinity of the borehole. Comprehensive inference based on combined x- and y-component responses indicates that the second-layer ore body intersecting the ore head is positioned in the borehole’s southeast direction and displays a gentle northwestward dip. Subsequent drilling operations on zk507 and zk508 confirmed this conclusion (Figure 9c), and ultimately, geological experts revised the geological profile of zk506. It is worth noting that, after the rock and mineral testing, the grade of the third abnormal body was downgraded in the subsequent compilation. This is consistent with the interpretation of the measured curve, meaning that the scale is large but the grade is relatively low. As a result, the z-component response curve only shows reactions in the early and middle stages and exhibits no abnormality in the late stages.

Based on the distribution of the inverted eddy currents (Figure 10), the anomaly at 370 m is identified as the main anomaly. It is situated in the southeast of the borehole, which is in accordance with the description in the geological logging of borehole zk506. Notably, following the actual inversion of the high-grade Ni17 ore body anomaly, the center of the anomaly was slightly uplifted, falling within the depth range of 360 m to 365 m. This indicates that the anomaly in the southeast direction exhibited an upward-trending pattern. Notably, due to the spatial separation between the firing position of the launch frame and the Ni17-1 ore body (Figure 7b), full excitation coupling with the ore body could not be achieved. Additionally, borehole zk506 was located at a significant distance from the Ni17-1 ore body, resulting in the Ni17-1-induced anomalies in the measured data being both small in magnitude and attenuated, thus rendering them difficult to identify.

## 5. Conclusions

This paper proposes an anomaly field extraction and equivalent eddy current inversion method based on layered-earth responses, enabling rapid quantitative inversion of BHTEM data. The correctness and applicability of this method are verified through theoretical calculation examples and borehole field measurements, providing a simple, rapid, and feasible inversion-interpretation approach for BHTEM data processing and interpretation and further improving the technical level of inversion interpretation. However, this method also has shortcomings and limitations. First, it has strong applicability to simple geological models, but there may be deviations in the inversion and interpretation of complex geological models. Therefore, in future research, advanced technologies such as machine learning can be used to implement 3D inversion to more accurately invert the electrical structure of subsurface media. Second, if high-conductivity layers or saturated aquifers exist in the shallow surface, they may act as interference bodies and mask ore body anomalies. Therefore, multi-parameter geophysical surveys may be an effective way to improve the credibility of interpretations.

## Figures and Tables

**Table 1:** Parameter list of algorithm verification mode.

	$\rho_{0}\;(\Omega \cdot m)$	$\rho_{1}\;(\Omega \cdot m)$	$h_{1}\;(m)$	$\rho_{2}\;(\Omega \cdot m)$	$h_{2}\;(m)$	$\rho_{3}\;(\Omega \cdot m)$
Uniform Half-space	10^8^	100	/	/	/	/
D-type	10^8^	500	50	50	/	/
G-type	10^8^	50	50	500	/	/
H-type	10^8^	500	50	50	10	5000
Q-type	10^8^	50	50	5000	10	500

$\rho_{0}$ is the resistivity of the air layer, with its resistivity set to 10^8^ Ω·m; $\rho_{i}$ and $h_{i}$ are the resistivity and layer thickness of the layer, respectively.

**Table 2:** Relative errors of two algorithms.

	Model	Uniform Half-Space	D-Type	G-Type	H-Type	Q-Type
Channel Number						
2		3.96%	2.07%	1.59%	2.49%	1.60%
3		1.10%	2.06%	1.58%	2.05%	1.58%
5		0.20%	1.57%	1.17%	0.94%	1.17%
7		0.55%	1.02%	0.60%	0.18%	0.60%
8		0.53%	0.73%	0.32%	0.40%	0.31%
10		0.23%	0.28%	0.27%	1.02%	0.28%
12		0.15%	0.13%	0.75%	1.47%	0.75%
14		0.40%	0.37%	1.06%	1.44%	1.06%
15		0.25%	0.19%	0.69%	1.22%	0.69%
17		0.54%	0.44%	1.00%	1.69%	1.00%
19		0.73%	0.64%	1.26%	1.94%	1.26%
20		1.04%	0.95%	1.46%	1.24%	1.45%
23		1.09%	1.02%	1.75%	1.64%	1.74%
24		0.99%	0.89%	1.11%	1.71%	1.10%
25		1.01%	0.95%	1.59%	1.19%	1.58%
26		1.04%	0.95%	1.17%	2.21%	1.18%
27		1.12%	1.03%	1.55%	0.99%	1.53%
28		1.02%	0.95%	1.27%	2.60%	1.27%

**Table 3:** Typical statistics of eddy current.

Parameter	ax (m)	ay (m)	az (m)	Dip (°)	DD (°)
	50.0	51.7	97.5	31.3	30.0

ax: x coordinate, ay: y coordinate, az: z coordinate, Dip: inclination, DD: rotation angle.

**Table 4:** The parameters of the layered-earth model.

Model	$\rho_{0}\;(\Omega \cdot m)$	$\rho_{1}\;(\Omega \cdot m)$
1	10^8^	300
2	10^8^	290
3	10^8^	310

**Table 5:** Statistics of regional electrical properties in east of Huangshan, Xinjiang (According to [36]).

Number	Rock (ore)	Samples	$\rho_{s}\;(\Omega \cdot m)$ Range	$\rho_{s}\;(\Omega \cdot m)$ Average	$\eta_{s}$ (%) Range	$\eta_{s}$ Average
1	Silicified marble	8	271–6099	2258.18	0.15–2.75	1.05
2	Biotite gneiss	2	292–3917	1069.46	1.54–2.09	1.79
3	Granite	11	369–11,656	1992.22	0.35–4.43	2.22
4	Gabbro	80	10–12,980	646	0.09–32.47	2.59
5	Peridotite	35	0–17,038	365	0.11–7.72	1.04
6	Mineralized peridotite	12	0.45–6875.4	88	10.99–50.9	28.38
7	Pyroxene diorite	6	162–4309	869	0.13–0.89	0.39
8	Pyroxenite	5	114–370	228	0.09–0.5	0.26
9	Tuffaceous schist	17	90–51,131	720	0.08–9.74	0.84
10	Diorite	13	198–13,387	1043	0.18–13.56	3.13
11	Norite	9	145–6283	1417	0.08–9.1	1.14

**Table 6:** Initial parameters of borehole zk506’s anomaly field inversion.

	Parameter	ax (m)	ay (m)	az (m)	R (m)	I (A)	Dip (°)	DD (°)
Eddy Current								
1		−10	−10	−345	4	0.001	0	0
2		40	−40	−362	15	0.005	20	−30
3		0	0	−390	8	0.0005	20	20

R: radius; I: current. The remaining parameters are consistent with those in Table 3.

**Table 7:** The average spatial position, dip, and rotation of the equivalent eddy current.

Eddy Current	ax (m)	ay (m)	az (m)	Dip (°)	DD (°)
1	−10.5	−10.5	−350	0	0
2	−39	−38	−361	20.8	−29.2
3	0	0	−401	20.1	20

The parameters are consistent with those in Table 3.

## Data Availability

Data associated with this research are available and can be obtained by contacting the corresponding author.
